# From Morphology to Biomarkers: Optical Coherence Tomography in Corneal Disease

**DOI:** 10.3390/diseases14090336

**Published:** 2026-09-14

**Authors:** Lanxing Fu, Yvonne H.-L. Luo, Nick Kopsachilis, Matteo Capobianco, Irene Gattazzo, Francesco Cappellani, Fabiana D’Esposito, Caterina Gagliano, Marco Zeppieri

**Affiliations:** 1 Department of Ophthalmology, East Kent Hospitals University NHS Foundation Trust, Kent CT1 3NG, UK; l.fu@nhs.net (L.F.);; 2 Eye Clinic, Catania University, Policlinico G. Rodolico, 95121 Catania, Italy; 3 Department of Ophthalmology, Ospedale Sant’Antonio, Azienda Ospedaliera, 35127 Padova, Italy; 4 Department of Medicine and Surgery, University of Enna “Kore”, 94100 Enna, Italy; 5 Mediterranean Foundation “G.B. Morgagni”, 95125 Catania, Italy; 6 Imperial College Ophthalmic Research Group (ICORG) Unit, Imperial College, London NW1 5QH, UK; 7 Department of Ophthalmology, University Hospital of Udine, 33100 Udine, Italy; 8 Department of Medicine, Surgery and Health Sciences, University of Trieste, 34129 Trieste, Italy

**Keywords:** optical coherence tomography, anterior segment OCT, cornea, keratoconus, corneal biomarkers, epithelial thickness mapping, corneal imaging, keratitis

## Abstract

Background: Optical coherence tomography (OCT) has been recognised as a non-invasive imaging modality capable of offering high-resolution visualisation of the corneal tissue. Objective: This review evaluates the role of OCT in chronic corneal disease, focusing on how descriptive imaging has progressed to quantitative imaging biomarkers with translational potential. Methods: A comprehensive review of the published literature on OCT imaging in corneal disease was conducted, emphasising structural features, quantitative parameters, and biomarkers. Results: OCT facilitates detailed visualisation of corneal microstructure, including variations in epithelial thickness, stromal reflectivity patterns, corneal thickness distribution, and interface abnormalities. These morphological observations are converted into quantitative measures that function as imaging biomarkers for disease severity, progression, and therapeutic effectiveness. In conditions such as keratoconus, chronic infectious keratitis, and corneal dystrophies, OCT-derived parameters offer valuable information for phenotyping and longitudinal monitoring. Additionally, advances in automated image analysis and artificial intelligence further increase the potential of OCT for biomarker discovery and evidence-based clinical decision-making. Conclusions: The development and validation of robust OCT-based biomarkers have the potential to enable earlier diagnosis, improved disease monitoring, and personalised treatment strategies. Future research that integrates quantitative imaging, computational analysis, and longitudinal clinical data will be essential to fully realise the translational potential of OCT in corneal disease management.

## 1. Introduction

The management of corneal disease increasingly depends on objective imaging techniques that supplement clinical examination. Although slit-lamp biomicroscopy remains the foundation of anterior segment assessment, its interpretation is subjective and may not accurately represent the depth, extent, or progression of structural abnormalities. Advances in anterior segment optical coherence tomography (AS-OCT) have transformed corneal imaging by enabling rapid, non-invasive, high-resolution visualisation of corneal tissue [1].

Since its introduction into clinical practice, OCT has evolved from a predominantly descriptive imaging modality into a quantitative platform capable of generating clinically meaningful biomarkers. Modern spectral-domain and swept-source systems provide detailed assessment of epithelial thickness, pachymetric distribution, stromal reflectivity, corneal oedema, and postoperative anatomy with excellent reproducibility [2,3,4]. These measurements support diagnosis, disease staging, progression monitoring, and evaluation of treatment response across a broad spectrum of corneal disorders.

Similar advances have been reported in infectious keratitis, corneal dystrophies, endothelial disease, and corneal surgery, where OCT provides structural information that may not be apparent on clinical examination alone. This review examines the evolution of OCT technology, the development of OCT-derived biomarkers, and the current applications of OCT in corneal disease. Automated image analysis and artificial intelligence are increasingly incorporated into OCT, supporting more detailed characterisation of corneal pathology. OCT now provides objective biomarkers that inform clinical decision-making and enhance patient management. Table 1 summarises the key studies.

This review is organised around the progression of AS-OCT from qualitative cross-sectional imaging to quantitative biomarker evaluation, rather than merely presenting an additional catalogue of AS-OCT findings in specific corneal illnesses. It focuses on factors that can be assessed over time, used in clinical decision-making, or combined with automated image analysis. The evaluation has also included current advancements in swept-source and ultra-high-resolution imaging, intraoperative OCT, and artificial intelligence.

## 2. Search Methodology

We conducted a literature search for our narrative review to identify papers on anterior segment optical coherence tomography (AS-OCT) in corneal pathology. We searched PubMed/MEDLINE, Scopus, and Web of Science from the beginning of their databases through [May 2026. Search queries have encompassed various combinations of terms such as ‘anterior segment optical coherence tomography’, ‘AS-OCT’, ‘cornea’, ‘corneal disease’, ‘keratoconus’, ‘epithelial thickness’, ‘pachymetry’, ‘keratitis’, ‘corneal infection’, ‘corneal dystrophy’, ‘Salzmann nodular degeneration’, ‘ocular surface squamous neoplasia’, ‘corneal endothelium’, ‘Fuchs endothelial corneal dystrophy’, ‘bullous keratopathy’, ‘corneal transplantation’, ‘keratoplasty’, ‘intraoperative OCT’, ‘artificial intelligence’, and ‘imaging biomarkers’. We considered original clinical trials, diagnostic investigations, observational research, pertinent case series, and reviews offering therapeutically significant insights on AS-OCT use in corneal pathology. Exclusions were made for publications that did not focus on corneal or anterior-segment OCT, studies without clinically pertinent imaging data, duplicate articles, conference abstracts with insufficient methodological details, and those for which appropriate data were unattainable. We examined reference lists of pertinent papers to identify additional studies. The evidence synthesis evaluated the study’s significance, design, methodological attributes, and relevance to clinical practice. This paper is a narrative review rather than a systematic review; therefore, we did not perform a formal meta-analysis or quantitative synthesis of risk of bias. The spatial performance of OCT has been described using quantitative specifications whenever these have been available, particularly axial resolution in tissue and acquisition speed, rather than subjective terms such as ‘good resolution’. Because these parameters vary according to wavelength, optical design, device generation, and manufacturer, the values reported should be interpreted as representative ranges rather than universal specifications.

## 3. Evolution of Anterior Segment Optical Coherence Tomography Technology

OCT was developed initially for imaging the retina. It has since been adapted for anterior segment applications, and now provides non-invasive, high-resolution cross-sectional visualisation of the cornea and surrounding ocular structures. Table 2 summarises how OCT imaging techniques have progressed and their applications.

### 3.1. Principles of OCT Imaging

OCT is based on low-coherence interferometry, a technique that measures the delay and intensity of backscattered light to generate high-resolution images of tissues [1]. The non-contact nature of OCT allows rapid, repeated imaging of the cornea without epithelial disruption. This makes OCT suitable for longitudinal monitoring and postoperative assessment.

### 3.2. Time-Domain OCT

The earliest anterior segment OCT systems utilised time-domain (TD) technology. These devices generated images by mechanically moving a reference mirror to measure reflected light from ocular tissues. Although TD-OCT represented a major advance over conventional examination techniques, acquisition speeds were relatively slow, and image resolution was limited [13].

Despite these limitations, early studies demonstrated the value of OCT for measuring corneal thickness, evaluating anterior chamber anatomy, and visualising corneal pathology [2,13]. TD-OCT established the basis for technological developments that would dramatically expand clinical applications.

### 3.3. Spectral-Domain OCT

Rather than relying on a moving reference mirror, SD-OCT simultaneously captures reflected light spanning multiple wavelengths and reconstructs images using Fourier transformation. This approach substantially increased acquisition speed while improving image resolution and signal quality.

Higher-resolution imaging resulted in more detailed visualisation of individual corneal layers, including the epithelium, Bowman’s layer, stroma, Descemet membrane, and endothelium. Subsequently, these advances led to epithelial thickness mapping, corneal pachymetric analysis, and detailed assessment of corneal microarchitecture [14].

The ability to generate reproducible quantitative measurements transformed OCT from a descriptive imaging modality into an important diagnostic and monitoring tool. Many of the biomarkers currently used in keratoconus, limbal stem cell deficiency, and postoperative corneal assessment were first established using SD-OCT technology [1].

### 3.4. Swept-Source OCT

Further improvements were achieved with the development of swept-source OCT (SS-OCT). Instead of using a broadband light source, SS-OCT uses a rapidly tunable laser that sweeps across a range of wavelengths. This technology allows faster image acquisition, deeper tissue penetration, and improved signal quality.

The longer wavelengths commonly used in SS-OCT systems penetrate opaque tissue more effectively than visible-light imaging modalities. Consequently, image quality can often be maintained in eyes with corneal scarring, oedema, or stromal opacification [5,6].

Modern SS-OCT devices can rapidly acquire high-density three-dimensional datasets, supporting comprehensive assessment of corneal thickness, epithelium, anterior chamber anatomy, and posterior corneal morphology. Commercial systems achieve axial resolutions of 5 to 10 µm and acquisition speeds of 30,000 to 70,000 A-scans per second [2,6].

### 3.5. Ultra-High-Resolution Optical Coherence Tomography

Ultra-high-resolution optical coherence tomography (UHR-OCT) uses wider-bandwidth light sources, resulting in axial resolutions near 1–3 µm in tissue, compared with the usual 5–10 µm range in other clinical OCT systems. However, specifications vary by device design and wavelength. This enhanced axial resolution allows for a more intricate imaging of the epithelium, Bowman’s layer, anterior stroma, and the boundaries between healthy and diseased tissue. UHR-OCT has therefore been examined for epithelial and subepithelial conditions, such as corneal dystrophies and degenerations, as well as ocular surface neoplasia, where accurate identification of pathology within specific corneal layers should enhance morphological assessment [8,9,10,11]. Nevertheless, enhanced spatial resolution does not inherently translate into improved clinical outcomes, and constraints such as limited accessibility, limited field of view in certain systems, acquisition prerequisites, and the absence of accepted quantitative benchmarks have hindered extensive routine use. UHR-OCT should be considered a supplementary imaging technique rather than a substitute for existing AS-OCT systems.

### 3.6. Comparison with Alternative Imaging Modalities

Corneal assessment has typically relied on slit-lamp biomicroscopy, ultrasound pachymetry, and Scheimpflug tomography. Each modality delivers valuable information, but OCT offers several specific advantages. Compared to slit-lamp examination, OCT provides objective cross-sectional imaging and allows accurate assessment of lesion depth, stromal involvement, and posterior corneal pathology. Quantitative measurements can be recorded and compared over time, thereby reducing reliance on subjective clinical estimates.

Unlike ultrasound pachymetry, OCT is noncontact and provides full corneal thickness maps rather than isolated point measurements. This improves patient comfort, eliminates risks associated with probe contact, and allows evaluation of the cornea’s regional thickness.

Scheimpflug imaging remains highly valuable for assessing corneal curvature, elevation, and tomography. However, image quality may be reduced in the presence of corneal opacity because visible light is scattered by the diseased tissue. OCT generally provides superior visualisation of individual corneal layers and performs better in eyes with significant stromal pathology [14,15]. Consequently, OCT and Scheimpflug imaging are best regarded as complementary rather than competing technologies [15].

## 4. OCT-Derived Biomarkers of Corneal Structure

The evolution of anterior segment optical coherence tomography (AS-OCT) has changed corneal imaging, moving from qualitative visualisation of tissue structure to quantitative assessment of structural biomarkers (Table 3). In corneal disease, biomarkers are objective imaging parameters that reflect pathological processes, disease severity, disease progression, or treatment response. By providing reproducible measurements, OCT has enabled more accurate diagnosis and more personalised approaches to patient management.

### 4.1. Pachymetric Biomarkers

Corneal thickness remains one of the most widely used structural biomarkers in ophthalmology. Although central corneal thickness (CCT) has traditionally been the focus of clinical assessment, modern OCT systems generate comprehensive pachymetric maps that provide information on regional thickness distribution of the cornea.

In healthy eyes, corneal thickness follows a predictable pattern, with gradual thickening from the centre toward the periphery. Many corneal diseases disrupt this normal architecture. Consequently, contemporary OCT assessment increasingly focuses on spatial thickness distribution and pachymetric progression rather than on a few central measurements.

Pachymetric analysis is valuable in ectatic disorders, where focal stromal thinning may precede clinically apparent changes in corneal curvature. Similarly, changes in corneal thickness provide functional indicators of disease activity in inflammatory conditions, endothelial dysfunction, and postoperative corneal oedema [1,2,3,4,5,6].

A major advantage of OCT-based pachymetry is its excellent repeatability [14]. Modern spectral-domain and swept-source systems provide highly reproducible measurements, enabling clinicians to distinguish true structural change from measurement variability and reliably monitor longitudinal change.

### 4.2. Epithelial Thickness Mapping

The corneal epithelium is a dynamic tissue that continuously remodels in response to changes in underlying stromal architecture [16]. As a result, epithelial thickness patterns often provide sensitive indicators of disease that may not be apparent when evaluating total corneal thickness alone. OCT allows separate analysis of epithelial and stromal layers, revealing compensatory epithelial responses that can mask underlying pathology. In ectatic disease, for example, epithelial thinning frequently occurs over areas of stromal protrusion, while adjacent regions demonstrate relative thickening. This results in a smoother anterior corneal surface but may conceal early structural abnormalities from conventional topographic analysis [17].

The diagnostic utility of epithelial mapping goes beyond keratoconus. Altered epithelial thickness profiles have been reported in limbal stem cell deficiency, dry eye disease, contact lens-related corneal changes, and corneal oedema [18,19,20,21]. Quantitative epithelial assessment therefore yields valuable information regarding ocular surface health and corneal homeostasis. Epithelial remodelling often precedes clinical signs, making epithelial thickness mapping a valuable OCT-derived marker for early disease detection.

### 4.3. Stromal Reflectivity and Tissue Characterisation

In addition to thickness measurements, OCT provides information regarding tissue reflectivity. Corneal reflectivity is influenced by collagen organisation, hydration status, inflammatory infiltration, fibrosis, and deposition of abnormal extracellular material. Changes in optical reflectivity therefore serve as indirect indicators of underlying tissue pathology.

In infectious and inflammatory conditions, stromal infiltrates typically appear as areas of increased reflectivity [6]. In corneal scars and dystrophies, abnormal tissue deposition produces characteristic hyperreflective patterns that assist diagnosis and monitoring [22]. Similarly, endothelial dysfunction and chronic oedema are associated with increased stromal reflectivity and disruption of normal corneal architecture. Reflectivity measurements are affected by imaging parameters and currently lack standardisation, but they provide opportunities for objective tissue characterisation.

### 4.4. Morphological Biomarkers

Many corneal disorders have characteristic structural features that can be visualised with OCT [2]. These morphological biomarkers often complement quantitative measurements and provide additional diagnostic information. Examples include epithelial thinning and stromal protrusion in keratoconus, stromal infiltrates in microbial keratitis, hyperreflective deposits in corneal dystrophies, Descemet membrane abnormalities in endothelial disease, and interface changes following corneal surgery [1,2,3,4,5,6,7]. These findings permit more precise localisation of pathology and contribute to disease classification and staging. Importantly, OCT allows these features to be documented objectively and monitored over time, providing clinicians with a detailed record of structural change that may not be captured by clinical examination alone.

High-resolution AS-OCT has taken on a significant role in evaluating ocular surface squamous neoplasia (OSSN). Distinctive epithelial attributes, such as epithelial hypertrophy, heightened reflectivity, and a demarcation between normal and pathological epithelium, may help identify diseases affecting the cornea and limbus [8,9,10,11]. Beyond the initial diagnosis, sequential OCT imaging can improve clinical assessment throughout therapy by documenting changes in epithelial structure and detecting ongoing or subclinical irregularities that may be difficult to observe with a slit lamp. These traits could be especially beneficial for assessing treatment efficacy and during follow-up monitoring. The progressive application of topical treatments for recurrent OSSN, particularly those including interferon, has underscored the importance of objective longitudinal evaluation of the ocular surface [23].

## 5. Keratoconus: The Prototypical OCT Biomarker Disease

Keratoconus represents the most extensively studied application of anterior segment optical coherence tomography (AS-OCT) in corneal disease and functions as a model for the development of OCT-derived biomarkers [5]. Characterised by progressive corneal thinning, protrusion, and irregular astigmatism, keratoconus may cause significant visual impairment if not detected and managed appropriately. The introduction of OCT has radically changed the approach to keratoconus by allowing detailed assessment of epithelial and stromal architecture, facilitating earlier diagnosis, more accurate disease staging, and objective monitoring of progression [24,25].

### 5.1. Early Detection and Subclinical Disease

One of the greatest clinical challenges in keratoconus management is identifying disease before substantial structural change and visual deterioration occur. This is particularly important in candidates for corneal refractive surgery, where undiagnosed keratoconus or forme fruste disease is a major risk factor for postoperative ectasia.

Traditional screening relies on corneal topography and tomography. However, structural abnormalities often precede detectable topographic changes. OCT has addressed this shortcoming by enabling layer-specific corneal analysis, uncovering subtle changes that may indicate early disease [5].

**Figure 1:**
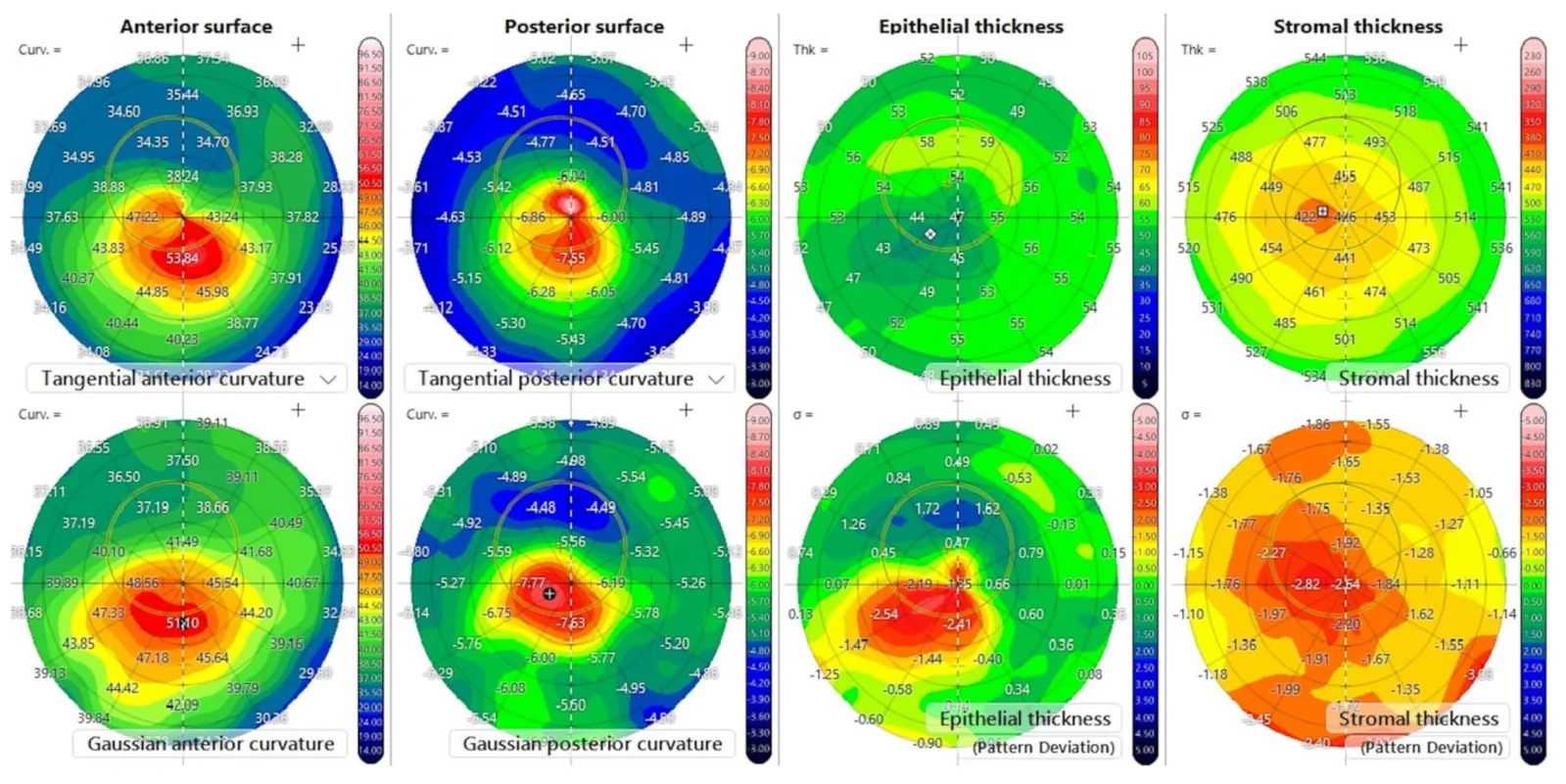
Characteristic pachymetric, epithelial maps, and stromal thickness patterns in keratoconus.

A significant contribution of OCT is delineating epithelial thickness patterns as preliminary imaging indicators for keratoconus [24]. Epithelial attenuation is frequently noted in the vicinity of stromal protrusion, while neighbouring regions may exhibit comparative epithelial hypertrophy (Figure 1). This spatial configuration is typically seen as a compensatory remodelling reaction that somewhat normalises the anterior corneal surface, potentially reducing the perceived severity of early stromal ectasia on standard anterior-surface topography. Nonetheless, the cellular and biomechanical mechanisms regulating epithelial redistribution in keratoconus remain poorly understood. Notable OCT characteristics include localised inferotemporal epithelial thinning, increased variability an asymmetric distribution in epithelial thickness, These irregularities can occur in eyes with mild or seemingly normal topographic results, potentially helping identify early or preclinical disease.

### 5.2. Pachymetric and Stromal Biomarkers

Progressive stromal thinning is a hallmark of keratoconus. Modern OCT systems generate detailed pachymetric maps that provide information beyond central corneal thickness alone. Analysis of the thinnest corneal point, pachymetric progression, and regional thickness distribution has significantly improved disease detection and staging.

Keratoconic corneas typically have localised inferotemporal thinning with abnormal progression of corneal thickness from the centre toward the periphery. These patterns can be quantified objectively and often provide greater diagnostic value than isolated thickness measurements. Combined evaluation of epithelial and stromal thickness maps further improves diagnostic performance by revealing compensatory epithelial changes that may obscure underlying stromal abnormalities [5]. The ability to assess individual corneal layers separately is a major advantage of OCT and has enhanced knowledge of the structural changes that occur throughout the disease course. Figure 1 shows the print out with the various parameters.

### 5.3. Monitoring Progression

Determining whether keratoconus is progressing is central to clinical management, especially when considering corneal cross-linking (CXL). Historically, progression has been assessed using changes in refraction, visual acuity, and keratometry. However, these parameters may fluctuate and often represent relatively late manifestations of disease progression.

OCT-derived biomarkers provide a more direct assessment of structural change. Serial evaluation of epithelial thickness patterns, pachymetric distribution, and stromal architecture permits clinicians to identify progression before significant visual deterioration occurs [17,25]. Progressive epithelial remodelling, increasing stromal thinning, and displacement of the thinnest corneal point may all indicate disease advancement. This is particularly important in younger patients, who are at higher risk of rapid progression and benefit most from early intervention.

### 5.4. Corneal Cross-Linking Assessment

Corneal cross-linking has become the standard treatment for halting keratoconus progression. OCT plays an important role in postoperative assessment by visualising the stromal demarcation line, which represents the transition between treated and untreated corneal tissue [26].

The demarcation line appears as a hyperreflective band within the anterior stroma and can be measured using OCT. Differences in demarcation depth have been observed among conventional, accelerated, and transepithelial cross-linking protocols, reflecting variations in treatment penetration [27]. OCT provides a non-invasive method for assessing treatment effects and comparing different CXL protocols.

### 5.5. Artificial Intelligence and Future Developments

The combination of artificial intelligence (AI) with OCT imaging represents a significant advance in the diagnosis of keratoconus. Machine-learning algorithms can analyse complex imaging patterns in epithelial thickness maps, pachymetric distributions, and elevation data, allowing automated disease detection and classification [28,29].

Several studies have reported excellent diagnostic performance with AI-based models, achieving accuracy in distinguishing normal corneas from keratoconic eyes. Emerging algorithms have also demonstrated potential to predict disease progression and identify patients most likely to benefit from early intervention. A recent example is the data-driven approach used by Kandakji et al. to identify subclinical keratoconus (SKC) from anterior segment OCT (MS-39) data across 25,816 scans from 5005 patients [30]. The authors employed a semi-supervised machine learning framework that combined principal component analysis and Gaussian mixture modelling to identify a high-risk subclinical keratoconus (SKC) phenotype using multidimensional anterior segment OCT-derived biomarkers. These SKC eyes were largely classified as normal by conventional ABCD staging, yet they showed significant asymmetry, a higher predicted risk of future corneal cross-linking, and faster progression to clinical keratoconus. Longitudinally, 72.7% met established progression criteria [30]. Their data showed that probabilistic, multidimensional analysis can detect high-risk keratoconus earlier than traditional threshold-based classification systems.

OCT is now a fundamental part of keratoconus management, complementing topography and tomography and providing unique information on corneal microarchitecture. The use of OCT-derived biomarkers in keratoconus has established a framework that is now applied to other corneal diseases, highlighting the broader role of quantitative imaging in anterior segment practice.

## 6. Infectious and Inflammatory Corneal Disease

Infectious and inflammatory corneal diseases remain major causes of ocular morbidity worldwide and can result in permanent visual impairment if not recognised and treated promptly. While slit-lamp biomicroscopy remains the cornerstone of diagnosis, anterior segment optical coherence tomography (AS-OCT) delivers valuable cross-sectional imaging of corneal architecture, enabling objective assessment of disease severity and treatment response [6,7].

### 6.1. Microbial Keratitis

Microbial keratitis is characterised by corneal infiltration, inflammation, and varying amounts of tissue destruction. AS-OCT cannot directly identify the causative pathogen; however, it provides detailed information on the structural consequences of infection.

A common OCT finding is stromal hyperreflectivity corresponding to inflammatory infiltration [6]. The depth and extent of these lesions can be measured objectively, allowing assessment of disease severity and monitoring over time. Associated corneal oedema appears as increased stromal thickness, while epithelial defects can be visualised as areas of discontinuity or surface irregularity.

**Figure 2:**
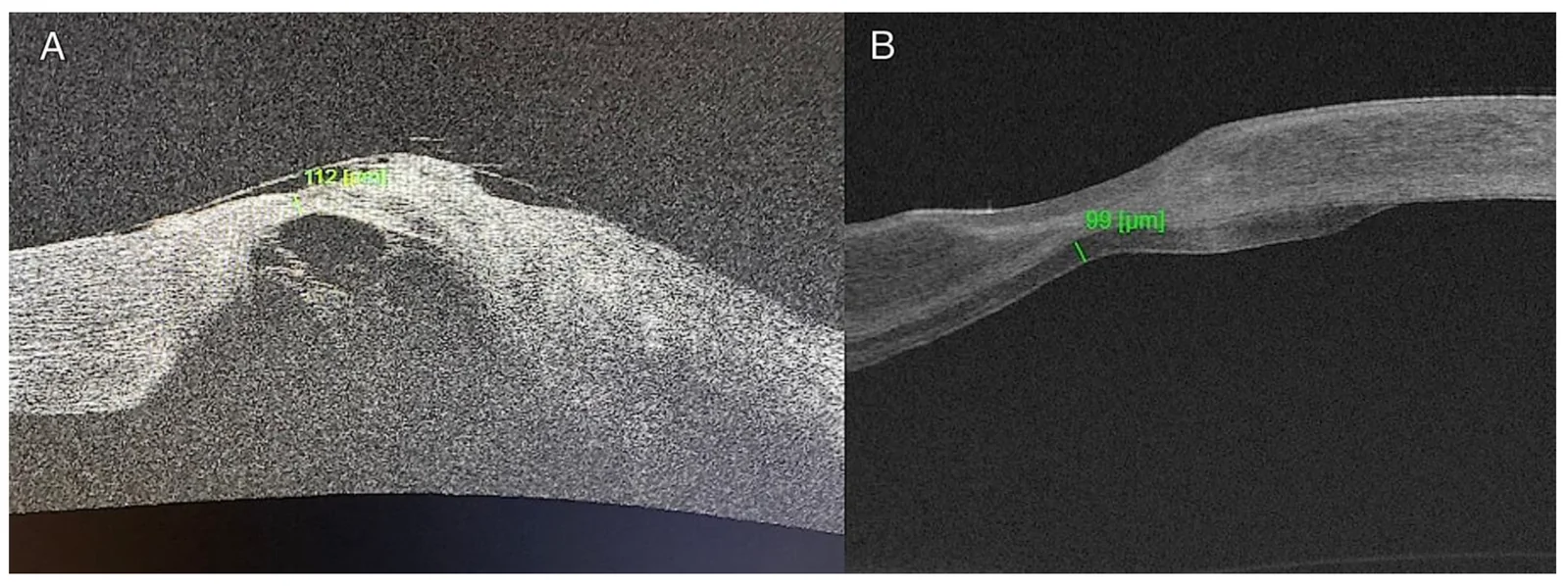
Optical coherence tomography scan of an impending corneal perforation with 112 µm descemetocele on the (left (**A**)) and after tectonic mini-DSEK (99 µm) repair (right; (**B**)).

One of the most clinically valuable applications of OCT is the evaluation of corneal thinning and melting (Figure 2). Progressive tissue loss can be difficult to quantify clinically, particularly in severe infections. Serial OCT imaging provides objective measurement of residual stromal thickness and can identify eyes at risk of perforation [7]. This information may influence decisions regarding hospital admission, surgical intervention, or escalation of antimicrobial therapy.

### 6.2. Fungal Keratitis

Fungal keratitis often poses diagnostic and therapeutic challenges caused by delayed presentation, deep stromal involvement, and prolonged treatment requirements. OCT provides important structural information that supports microbiological and clinical assessment.

Typical findings include dense hyperreflective stromal infiltrates associated with surrounding oedema. Areas of stromal necrosis may appear as intrastromal cystic spaces or zones of tissue loss [31]. Although not specific to fungal infection, these features often indicate severe disease and may help differentiate fungal keratitis from less destructive inflammatory processes.

Retrocorneal plaques are another notable OCT finding [32]. These hyperreflective structures located posterior to the cornea may indicate deep fungal invasion and are associated with a poorer prognosis. OCT can assist in monitoring changes in infiltrate depth and corneal architecture during treatment and may aid surgical planning when therapeutic keratoplasty is required.

### 6.3. Acanthamoeba and Viral Keratitis

Acanthamoeba keratitis (AK) is frequently associated with delayed diagnosis because early clinical findings may be subtle. OCT has contributed toward improved recognition of characteristic structural features, particularly radial keratoneuritis [7]. This appears as hyperreflective linear bands extending from the subepithelial region into the anterior or mid-stroma and corresponds to inflammation of the corneal nerves.

As disease progresses, OCT may reveal stromal infiltration, tissue loss, and progressive corneal thinning. Although these signs are not unique to AK, they provide objective markers for tracking treatment response during what is often a prolonged clinical course [33].

In herpetic keratitis, OCT findings vary according to the disease subtype [6,7]. Epithelial disease typically produces superficial hyperreflective lesions and epithelial irregularity, whereas stromal keratitis is associated with bigger hyperreflective changes and corneal oedema. In disciform keratitis, OCT allows objective quantification of corneal swelling and can be used to monitor resolution during treatment.

### 6.4. Corneal Scarring and Visual Prognosis

Corneal scarring is a common consequence of severe infection and inflammation and is still a major determinant of long-term visual outcome. Clinical assessment of scar depth and extent can be challenging, particularly in irregular or densely opaque corneas.

**Figure 3:**
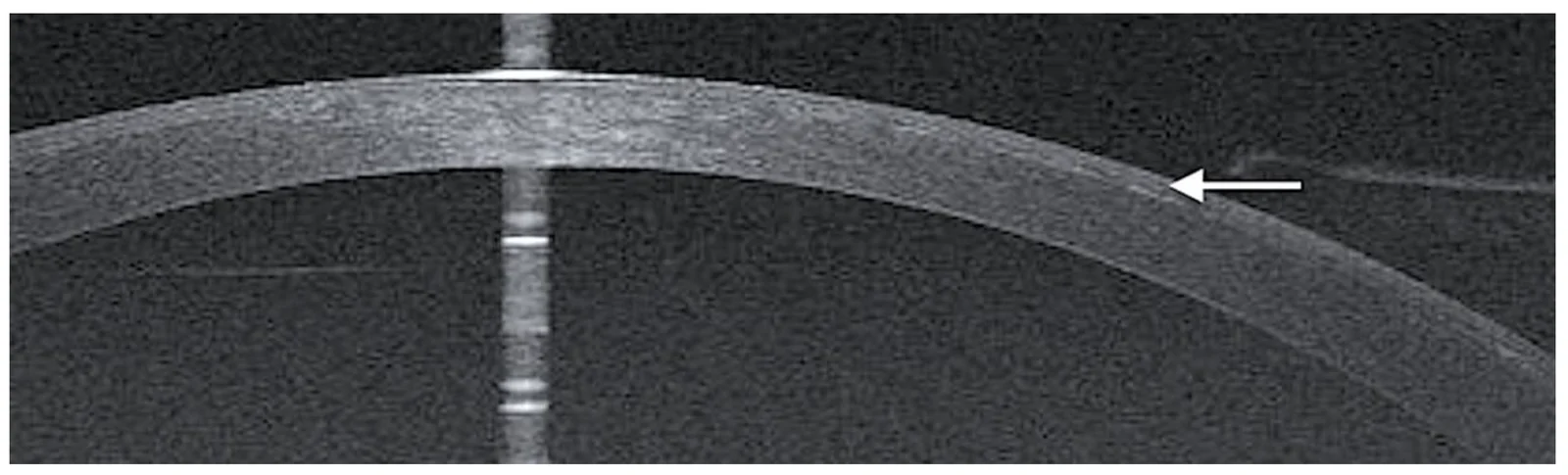
Optical coherence tomography scan of the cornea post laser in situ keratomileusis (LASIK) surgery; the white arrow indicates the location of the LASIK flap [33].

AS-OCT allows detailed characterisation of scar morphology, including depth, thickness, and stromal involvement. Scar tissue typically appears as areas of increased reflectivity within the stroma, for example, after laser refractive surgery (Figure 3). Sometimes it can be associated with architectural distortion and localised thinning. Quantitative measurements may help predict visual outcomes and identify patients who may benefit from surgical rehabilitation [33].

The ability to objectively assess scar characteristics is also valuable when planning procedures such as phototherapeutic keratectomy, deep anterior lamellar keratoplasty, or penetrating keratoplasty. OCT provides information that supports both diagnosis and long-term management. Although many disorders show characteristic slit-lamp findings, clinical examination alone may not accurately determine the depth, extent, or distribution of pathological changes [34].

## 7. Corneal Dystrophies and Degenerations

Corneal dystrophies and degenerative disorders comprise a diverse group of conditions characterised by progressive structural alterations affecting specific corneal layers. Although many disorders show characteristic slit-lamp findings, clinical examination alone may not accurately determine the depth, extent, or distribution of pathological changes [34].

### 7.1. Stromal Corneal Dystrophies

In stromal corneal dystrophies, there is abnormal deposition of extracellular material within the corneal stroma. OCT provides valuable information regarding the depth, distribution, and extent of these deposits, often complementing clinical examination.

In granular corneal dystrophy, deposits appear as discrete hyperreflective stromal opacities separated by relatively unaffected tissue. Lattice corneal dystrophy has characteristic linear hyperreflective lesions corresponding to amyloid deposition, while macular corneal dystrophy is associated with diffuse stromal reflectivity and widespread structural disorganisation [35]. These imaging features not only aid diagnosis but also help determine the depth of involvement and suitability for interventions such as phototherapeutic keratectomy (PTK), deep anterior lamellar keratoplasty (DALK), or penetrating keratoplasty.

### 7.2. Schnyder Corneal Dystrophy

Schnyder corneal dystrophy is a rare inherited disorder characterised by progressive deposition of cholesterol and phospholipids within the cornea. Clinical diagnosis may be challenging because crystalline deposits are absent in a substantial proportion of patients [22]. AS-OCT typically demonstrates hyperreflective bands or plaques within the anterior stroma corresponding to lipid accumulation. These findings may be present even when clinical examination is inconclusive, making OCT a valuable component of multimodal assessment.

### 7.3. Salzmann Nodular Degeneration

Salzmann nodular degeneration is a common non-inflammatory degenerative condition characterised by elevated subepithelial nodules. Although the diagnosis is often apparent clinically, OCT provides additional information regarding lesion morphology, depth, and involvement of the anterior corneal layers. Characteristic findings include localised hyperreflective nodules associated with disruption or replacement of Bowman’s layer and variable extension into the anterior stroma [36]. This anatomical delineation can complement slit-lamp examination and support surgical planning. In patients undergoing superficial keratectomy, intraoperative OCT has also been used to provide real-time assessment of lesion depth and the underlying corneal planes, facilitating controlled removal of the nodular tissue [37]. OCT can be used when clinical findings are atypical or when differentiation from other anterior corneal lesions is required [34].

### 7.4. Quantitative Assessment and Surgical Planning

One of the advantages of OCT in corneal dystrophies and degenerations is its ability to objectively quantify structural abnormalities. Measurements of lesion depth, stromal involvement, corneal thickness, and tissue reflectivity provide reproducible biomarkers that can be monitored over time.

This information is valuable when planning the timing of any surgical intervention. The depth and distribution of pathology often determine the most appropriate procedure. Superficial lesions may be managed with keratectomy or PTK, while deeper stromal involvement may require lamellar or penetrating keratoplasty [38]. OCT-derived measurements may help predict surgical success in procedures such as DALK and DMEK by identifying structural factors associated with favourable outcomes [12,38].

## 8. Endothelial Disease and Corneal Oedema

Corneal endothelial dysfunction is a major cause of visual impairment and a common indication for corneal transplantation worldwide. Failure of the endothelial pump mechanism results in progressive stromal hydration, loss of corneal transparency, and deterioration of visual quality. While slit-lamp biomicroscopy remains essential for clinical assessment, anterior segment optical coherence tomography (AS-OCT) has become increasingly important for evaluating the structural consequences of endothelial disease and monitoring treatment outcomes [39].

The principal strength of OCT lies in its ability to provide objective, reproducible measurements of corneal architecture. Beyond simple pachymetry, OCT enables assessment of posterior corneal morphology, stromal reflectivity, epithelial changes, and postoperative graft status [1,40,41]. These parameters increasingly serve as quantitative biomarkers of disease severity and progression.

### 8.1. Fuchs Endothelial Corneal Dystrophy

Fuchs endothelial corneal dystrophy (FECD) is a degenerative condition of the corneal endothelium marked by the formation of corneal guttata, atypical extracellular matrix accumulation at the Descemet membrane, gradual endothelial cell impairment and attrition, and, in later stages, compromised endothelial pump efficacy [39]. Deterioration of endothelial equilibrium leads to stromal and later epithelial swelling, with persistent conditions possibly causing subepithelial scarring and diminished visual acuity. OCT offers supplementary structural insights across this illness range. Early disease may demonstrate subtle posterior corneal irregularity associated with guttae, while more advanced disease is characterised by progressive stromal thickening and increased corneal reflectivity [39]. Importantly, OCT can identify structural evidence of chronic oedema, including anterior stromal fibrosis and subepithelial scarring, which may adversely affect visual recovery even after successful endothelial transplantation [39,40,41].

Corneal oedema is the hallmark of endothelial dysfunction [39]. Quantitative pachymetry provides an objective measure of stromal hydration and enables precise monitoring of disease progression. However, increasing evidence suggests that corneal thickness alone does not fully reflect disease severity. Some patients exhibit significant visual symptoms despite relatively modest increases in pachymetry, while others maintain functional vision despite substantial stromal thickening [40]. Consequently, contemporary OCT assessment increasingly incorporates evaluation of corneal architecture in addition to thickness measurements.

Bullous keratopathy (BK) is linked to severe endothelial dysfunction and can result from Fuchs’ endothelial corneal dystrophy (FECD), intraocular procedures, or eye injuries [41]. The condition is marked by stromal and epithelial swelling, epithelial blister formation, reduced vision, and ocular discomfort. AS-OCT examinations have revealed augmented corneal thickness alongside modifications in both anterior and posterior corneal geometry in eyes affected by BK [40]. These structural changes may lead to atypical astigmatism and reduced visual performance. Significantly, pachymetric assessments must be analysed alongside other OCT-derived structural characteristics, as corneal thickness alone may not adequately reflect the severity or duration of endothelial impairment. Serial OCT imaging can thus offer an impartial method for tracking alterations in corneal moisture and structure over time.

### 8.2. Descemet Membrane Detachment

Descemet membrane detachment (DMD) is a cause of persistent postoperative corneal oedema and may occur after cataract surgery, endothelial keratoplasty, glaucoma surgery, or ocular trauma. Small detachments can be difficult to visualise clinically, especially when significant corneal oedema is also present.

AS-OCT has become the preferred imaging modality for diagnosis and follow-up. Cross-sectional imaging allows precise visualisation of the detached Descemet membrane, measurement of the separation distance, and assessment of the detachment configuration [42]. These findings directly influence management decisions, helping clinicians determine whether observation, intracameral gas injection, or surgical intervention is required. Serial imaging also provides objective confirmation of membrane reattachment and resolution of associated oedema.

**Figure 4:**
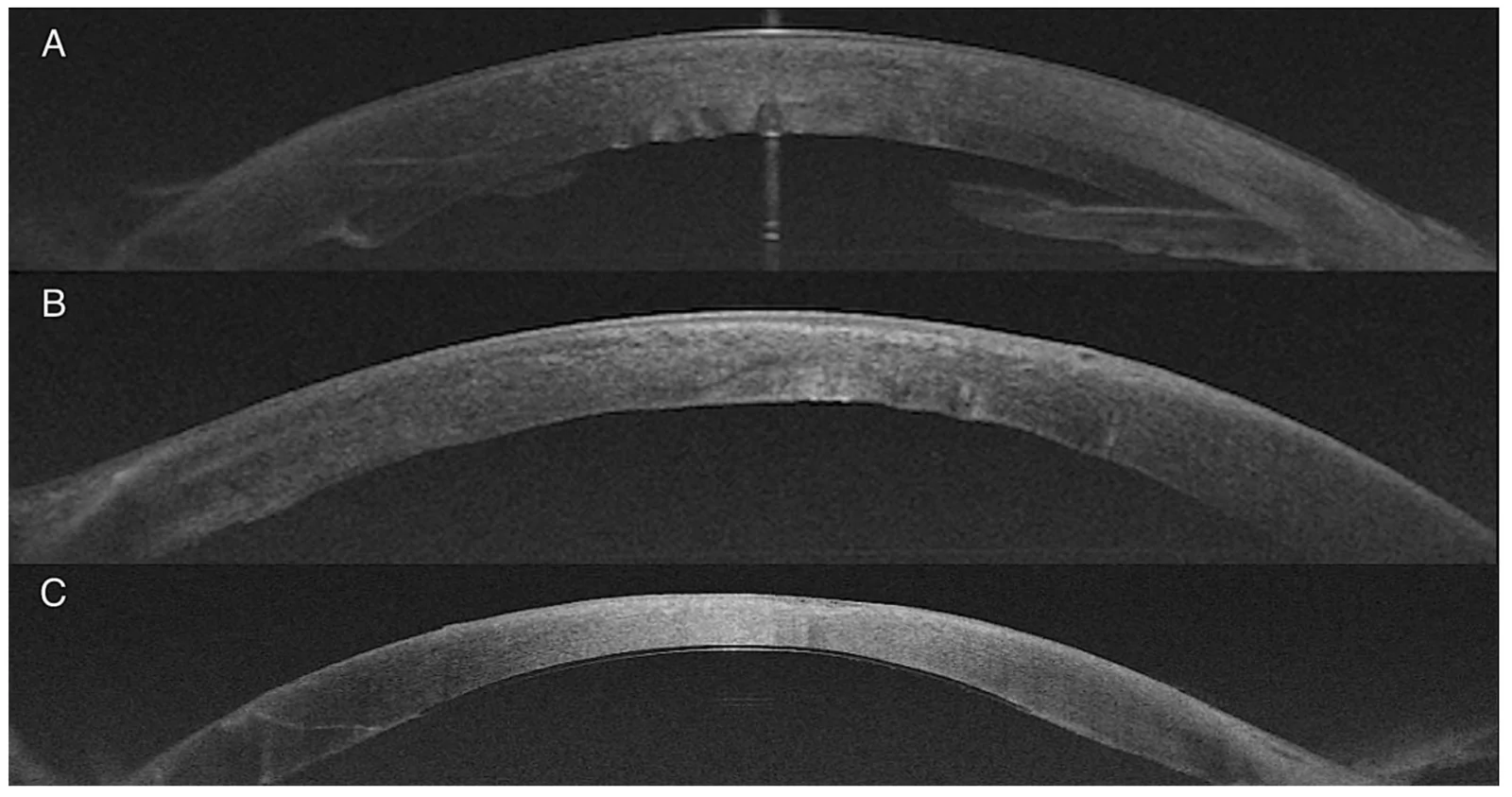
Optical coherence tomography scans demonstrating oedema in (**A**) Early graft failure following Descemet stripping endothelial keratoplasty (DSEK), (**B**) one day postoperative appearance following Descemet membrane endothelial keratoplasty (DMEK), (**C**) after EndoArt implantation.

Endothelial keratoplasty procedures, including Descemet stripping automated endothelial keratoplasty (DSAEK) and Descemet membrane endothelial keratoplasty (DMEK), have transformed the management of endothelial disease. OCT plays an important role throughout the perioperative period (Figure 4).

Preoperatively, OCT assesses corneal thickness, stromal fibrosis, and posterior corneal morphology. Postoperatively, it evaluates graft attachment, interface integrity, residual interface fluid, and corneal deturgescence. After corneal transplantation, OCT enables assessment of graft-host interfaces, graft attachment, and changes in corneal thickness.

In endothelial keratoplasty, subtle graft detachments and residual interface fluid can be identified with greater sensitivity than slit-lamp examination, allowing earlier intervention if necessary [41,42]. Similarly, OCT is valuable for diagnosing and following Descemet membrane detachment after cataract surgery and other anterior segment procedures [41].

## 9. OCT in Corneal Surgery

AS-OCT can guide the selection of surgical procedures based on the depth of pathology. Examples include epithelial debridement for pathologies limited to the epithelium and Bowman’s layer, phototherapeutic keratectomy for anterior stroma involvement, and deep lamellar keratoplasty for posterior stromal disease [43]. Analysis of both the anterior and posterior components of lamellar surgeries is possible, detecting interfaces after DSAEK, deep anterior lamellar keratoplasty, and femtosecond laser-enabled keratoplasty [44].

### Intraoperative OCT

The introduction of microscope-integrated intraoperative OCT (iOCT) has expanded the role of OCT beyond diagnosis and planning. Real-time imaging during surgery allows visualisation of tissue planes that may not be visible through conventional surgical microscopy [43,44,45].

In deep anterior lamellar keratoplasty (DALK), iOCT facilitates assessment of dissection depth and the formation of a big bubble, helping to minimise the risk of Descemet membrane perforation [44]. During endothelial keratoplasty procedures such as Descemet stripping automated endothelial keratoplasty (DSAEK) and Descemet membrane endothelial keratoplasty (DMEK), iOCT allows the evaluation of graft orientation, attachment, and residual interface fluid [45]. By improving intraoperative visualisation of surgical anatomy, iOCT enhances procedural precision and may reduce intraoperative complications.

The main limitations are the equipment costs, the learning curve for image interpretation, workflow integration, and a limited field of view. Current systems may also lack sufficient resolution to capture some microstructural details, and there are no standardised surgical protocols.

## 10. Artificial Intelligence and Future Directions

The rapid expansion of anterior segment optical coherence tomography (AS-OCT) has generated increasingly large and complex imaging datasets that extend beyond the capacity of traditional manual interpretation. Recent advances in artificial intelligence (AI), machine learning, and deep learning have therefore emerged as important developments in corneal imaging, offering opportunities to improve diagnostic accuracy, automate image analysis, and facilitate personalised patient care [28].

One of the earliest applications of AI in corneal OCT has been automated image segmentation. Accurate identification of corneal boundaries and individual tissue layers is fundamental for generating reliable measurements of epithelial thickness, stromal architecture, and corneal curvature [46].

Manual segmentation is time-consuming and subject to observer variability, particularly in diseased corneas where anatomical boundaries may be distorted. Machine-learning algorithms can automate this process, providing rapid and reproducible measurements while reducing operator dependence. Automated segmentation is especially valuable in keratoconus, corneal dystrophies, and postoperative corneas, where structural irregularity can challenge current software algorithms.

### 10.1. Disease Detection and Classification

Among corneal disorders, keratoconus has been the principal focus of AI-based OCT research. Deep-learning models trained on epithelial thickness maps, pachymetric distributions, and elevation data have demonstrated excellent performance in distinguishing normal corneas from keratoconic eyes. Several studies have reported diagnostic accuracies exceeding those achieved using individual imaging parameters alone [46,47].

Importantly, AI systems may improve the detection of subclinical and forme fruste keratoconus, in which structural abnormalities are subtle and difficult to identify with traditional approaches [47]. Earlier recognition of disease could enhance screening for refractive surgery and facilitate timely intervention before significant visual deterioration occurs.

Beyond keratoconus, AI-assisted analysis is being explored in infectious keratitis, corneal dystrophies, endothelial disease, and postoperative corneal assessment [46,47]. These applications remain in the early stages of development but highlight the broad potential of combining OCT imaging with computational analysis.

### 10.2. Predicting Disease Progression

A particularly promising application of AI is predicting disease progression. Current clinical management often relies on identifying structural changes after they have occurred. Predictive models can identify patients at greatest risk [28].

In keratoconus, machine-learning algorithms incorporating OCT-derived biomarkers, demographic variables, and clinical parameters have demonstrated encouraging predictive performance [30]. Such models may eventually help clinicians determine the optimal timing of corneal cross-linking and tailor follow-up intervals based on individual risk profiles. Similar approaches may prove valuable in endothelial disease, postoperative graft monitoring, and inflammatory corneal disorders, where earlier identification of high-risk patients could improve outcomes.

### 10.3. Multimodal Imaging and Precision Corneal Care

The future of corneal imaging is likely to extend beyond OCT alone. Increasing interest has focused on integrating OCT data with information obtained from corneal topography, Scheimpflug tomography, biomechanics, confocal microscopy, and genetic testing.

Combining multiple data sources can provide more comprehensive characterisation of disease phenotypes and improve diagnostic performance. Integrated risk scores based on multiple biomarkers may support personalised assessment of diagnosis, progression risk, and treatment response. This approach supports the trend toward precision medicine.

## 11. Conclusions

The utility of OCT also extends to infectious and inflammatory keratitis, corneal dystrophies, endothelial dysfunction, and postoperative assessment. Quantifying corneal thickness, lesion depth, tissue reflectivity, and structural changes over time has supported more individualised management.

Variability between imaging platforms, acquisition protocols, and segmentation algorithms limits comparability between studies and clinical settings. Many AI models have been developed using small datasets from single centres, which limits generalisability across populations and imaging platforms. Overfitting can occur when a model learns dataset-specific characteristics rather than true disease patterns, reducing reliability in new patients.

Combining OCT-derived biomarkers with multimodal imaging data may enable more comprehensive disease phenotyping. This would improve prediction of disease progression and treatment response, and support precision medicine. External validation, standardisation of imaging protocols, and transparent reporting of algorithm performance are required before widespread clinical adoption. Regulatory considerations and clinician oversight are necessary to ensure safe and effective integration into clinical practice.

## Figures and Tables

**Table 1:** Key Studies Evaluating Optical Coherence Tomography in Anterior Segment Disease.

Citation	Study Design	Sample Size	OCT Device	Key Findings	Strengths/Limitations	Level of Evidence
Li et al., 2012. [3]	Prospective case–control	Normal (76 eyes) and keratoconic (35) eyes	Fourier-domain OCT	Demonstrated characteristic epithelial thinning over the cone and compensatory annular thickening in keratoconus. Improved detection of early ectasia.	First large OCT epithelial mapping study.	Level II
Zhu et al., 2013. [4]	Prospective observational	Asymmetric keratoconus eyes	AS-OCT	Showed epithelial thickness variability differentiates clinically normal fellow eyes from keratoconic eyes.	Useful for subclinical disease; relatively small cohort.	Level II
Yang et al., 2021. [5]	Diagnostic accuracy study	176 eyes	Spectral-domain OCT	OCT epithelial and pachymetric maps improved detection of early and forme-fruste keratoconus.	Included subclinical disease; device-specific algorithm.	Level II
Soliman et al., 2013. [6]	Prospective observational	20 eyes	Spectral-domain AS-OCT	Identified characteristic OCT patterns in bacterial and fungal keratitis, including stromal necrotic cystic spaces in fungal infection.	Histopathological correlation; small sample size.	Level III
Abdelghany et al., 2021. [7]	Narrative review	Multiple studies	Various AS-OCT systems	Demonstrated utility of AS-OCT for infiltrate depth, corneal thinning, perforation risk, and treatment monitoring.	Comprehensive review; not primary evidence.	Level V
Thomas et al., 2014. [8]	Review of published studies	Multiple studies	UHR-OCT	Established thickened hyperreflective epithelium with abrupt transition as a hallmark OCT feature of OSSN.	Seminal review; lacks prospective validation.	Level V
Atallah et al., 2017. [9]	Retrospective diagnostic study	16 patients, 12 confirmed OSSN	High-resolution OCT	HR-OCT improved differentiation of OSSN from benign ocular surface lesions.	Histopathologic comparison; retrospective design.	Level III
Singh et al., 2018. [10]	Prospective observational	17 patients	HR-OCT	Characterised imaging features of intraepithelial and invasive OSSN.	Prospective design; relatively small sample.	Level II
Karp et al., 2019. [11]	Prospective cohort	8 OSSN patients undergoing surgery	HR-OCT	Demonstrated HR-OCT can identify residual subclinical tumour and guide treatment endpoints.	Strong clinical applicability; single disease focus.	Level II
Patel et al., 2020. [12]	Prospective case series	100 eyes	Intraoperative OCT	High reliability of iOCT for DMEK orientation with low rebubbling and graft failure rates.	Useful real-world evaluation of both experienced and novice surgeons, but no comparator arm.	Level IV

The Oxford Centre for Evidence-Based Medicine (OCEBM) Levels of Evidence framework categorises levels of evidence, primarily based on the diagnostic or monitoring questions each study addresses and its study design. (OCEBM Evidence Levels Working Group. The Oxford Levels of Evidence from 2011. Oxford Centre for Evidence-Based Medicine at the University of Oxford.).

**Table 2:** The evolution of OCT imaging techniques and their real-world applications [1,2,3,4,5,6,7].

Feature	Time-Domain OCT (TD-OCT)	Spectral-Domain OCT (SD-OCT)	Swept-Source OCT (SS-OCT)
HOW IT WORKS	Measures echo time delay as the reference mirror moves. Depth profile obtained point-by-point.	Measures the interference spectrum at once and uses Fourier transformation to obtain depth profile.	Sweeps wavelength over time; depth profile retrieved from interference signal vs. wavelength.
AXIAL RESOLUTION	10–15 μm (Lower resolution)	3–7 μm (Higher resolution)	5–10 μm (Good resolution)
ACQUISITION SPEED	~400–2000 A-scans/s (Slow)	~20,000–100,000+ A-scans/s (Fast)	~50,000–400,000+ A-scans/s (Very Fast)
SENSITIVITY	Lower (Limited by mechanical movement)	Higher (No moving parts in detection path)	Highest (Higher power at longer wavelengths + no moving parts)
PENETRATION DEPTH	~1–2 mm (Limited)	~2–3 mm	~3–5+ mm (Deepest)
WAVELENGTH RANGE	~800–900 nm	~800–900 nm	~1050 nm (or 1300 nm)
ADVANTAGES	Simple and low cost; Mature technology	High speed; High sensitivity; High resolution	Deep penetration; Highest speed and sensitivity; Less sensitivity roll-off with depth
LIMITATIONS	Slow acquisition; Lower sensitivity; Lower resolution	Limited penetration depth; More sensitivity roll-off with depth; Spectrometer size/cost	Higher system cost; More complex laser source
BEST SUITED FOR	Educational and research setups where cost is critical	General ophthalmic imaging, retina, anterior segment	Able to image deeper structures, choroid, posterior segment, and beyond, through diseased cornea

**Table 3:** A summary of AS-OCT applications, diagnostic/monitoring roles, and limitations in various corneal conditions.

Disease/Condition	Key OCT Signs (Abridged)	Diagnostic Role	Monitoring Role	Surgical Planning Role	Limitations
OSSN	Hyper-reflective thickened epithelium; abrupt transition; cystoid spaces	Differentiate malignant vs. benign lesions	Detect residual disease	Guide treatment initiation/cessation	Limited assessment of deeper invasion
LSCD	Epithelial thinning; loss of palisades of Vogt	Confirm diagnosis	Track epithelial recovery	Guide SLET vs. keratoplasty	May require OCTA differentiation
INFECTIVE KERATITIS	Epithelial defect; stromal infiltrate	Define ulcer depth	Monitor healing	Guide timing of intervention	Dense infiltrates obscure depth
KERATOCONUS	Central thinning; annular thickening	Early detection/risk stratification	Track progression	Guide CXL/Intacs planning	Not entirely specific
CORNEAL DYSTROPHIES	Layer-specific deposits	Classification/severity grading	Document progression	Guide keratoplasty choice	Overlap among dystrophies
CONGENITAL CORNEAL DISORDERS	Posterior defects; stromal thickening	Define structural abnormalities	Assess progression	Determine feasibility of surgery	Interpretation challenging
PTERYGIUM/PINGUECULA	Subepithelial hyper-reflective mass	Differentiate lesions	Monitor growth	Define excision extent	Limited deep scleral resolution
CORNEAL TRAUMA	Descemet detachment; wound gape	Detect occult injury	Monitor healing	Guide repair strategy	Reduced quality with oedema/haemorrhage
CORNEAL FOREIGN BODY	Hyper-reflective anterior border	Determine depth/location	Limited role	Guide removal approach	Posterior extent obscured
DRY EYE DISEASE	Reduced tear meniscus	Adjunct diagnostic tool	Monitor therapy response	—	Metrics affected by environment/blink
OCULAR CHEMICAL BURNS (OCTA)	Reduced limbal vessel density	Assess limbal ischemia	Serial monitoring	Guide early interventions	Cannot assess leakage
CORNEAL NEOVASCULARISATION (OCTA)	Abnormal vascular network	Detect subclinical NV	Monitor anti-angiogenic therapy	Pre-op vascular mapping	Limited standardisation
REFRACTIVE SURGERY	Flap thickness; interface fluid	Detect complications	Monitor healing	Essential for planning	Post-op haze/oedema affects interpretation
CORNEAL TRANSPLANTATION	Graft detachment/interface gap	Detect early complications	Monitor graft survival	Critical for peri-operative planning	Limited intra-op use without integrated OCT
SCLEROCORNEA/MPS	Diffuse stromal hyper-reflectivity	Support diagnosis	Monitor progression	Support planning	Overlap with other opacifying disorders
CORNEAL ULCERS/THINNING DISORDERS	Localised stromal thinning	Identify structural compromise	Track healing	Determine need for surgery	Limited specificity
ANIMAL/EXPERIMENTAL MODELS	Reflectivity ratio; wound depth	Research diagnostics	Track outcomes	Experimental planning	Limited clinical translation

## Data Availability

No new data were created or analysed in this study. Data sharing is not applicable to this article.
